# Comparison of functional activation responses from the auditory cortex derived using multi-distance frequency domain and continuous wave near-infrared spectroscopy

**DOI:** 10.1117/1.NPh.8.4.045004

**Published:** 2021-12-15

**Authors:** Penaz Parveen Sultana Mohammad, Sittiprapa Isarangura, Ann Eddins, Ashwin B. Parthasarathy

**Affiliations:** a University of South Florida, Department of Electrical Engineering, Tampa, Florida, United States; b University of South Florida, Department of Communication Sciences and Disorders, Tampa, Florida, United States

**Keywords:** near-infrared spectroscopy (NIRS), frequency domain measurements, continuous wave-NIRS, functional activation, functional near-infrared spectroscopy, auditory stimulation

## Abstract

**Significance:** Quantitative measurements of cerebral hemodynamic changes due to functional activation are widely accomplished with commercial continuous wave (CW-NIRS) instruments despite the availability of the more rigorous multi-distance frequency domain (FD-NIRS) approach. A direct comparison of the two approaches to functional near-infrared spectroscopy can help in the interpretation of optical data and guide implementations of diffuse optical instruments for measuring functional activation.

**Aim:** We explore the differences between CW-NIRS and multi-distance FD-NIRS by comparing measurements of functional activation in the human auditory cortex.

**Approach:** Functional activation of the human auditory cortex was measured using a commercial frequency domain near-infrared spectroscopy instrument for 70 dB sound pressure level broadband noise and pure tone (1000 Hz) stimuli. Changes in tissue oxygenation were calculated using the modified Beer–Lambert law (CW-NIRS approach) and the photon diffusion equation (FD-NIRS approach).

**Results:** Changes in oxygenated hemoglobin measured with the multi-distance FD-NIRS approach were about twice as large as those measured with the CW-NIRS approach. A finite-element simulation of the functional activation problem was performed to demonstrate that tissue oxygenation changes measured with the CW-NIRS approach is more accurate than that with multi-distance FD-NIRS.

**Conclusions:** Multi-distance FD-NIRS approaches tend to overestimate functional activation effects, in part due to partial volume effects.

## 1 Introduction

Over the last decade, functional near-infrared spectroscopy (fNIRS) has emerged as a reliable non-invasive method for monitoring cortical activity in the brain.^1^^–^^3^ fNIRS has been used for a variety of applications such as to study neuronal activation of brain circuits,^4^^–^^6^ to image functional/resting state connectivity of the human brain,^4^^,^^7^^,^^8^ to examine and characterize cognitive behavior,^9^^–^^11^ and to study/implement brain–computer interfaces.^12^^–^^14^ fNIRS instruments are especially useful for characterizing functional hemodynamic changes associated with the auditory system. It is generally difficult to measure cerebral activity in response to activations of the auditory cortex with clinical imaging modalities such as x-ray computed tomography or magnetic resonance imaging because instrument sounds increase background noise, which could corrupt the careful presentation of auditory stimuli to subjects and thereby significantly bias the results of an experiment. In part, because of these advantages, several recent studies^7^^,^^15^^–^^17^ have used commercial fNIRS instruments to characterize functional stimulation of auditory cortex in humans. For example, Chen et al.^7^ measured the hemodynamic responses from the auditory cortex for 440 and 554 Hz pure tones and a 1000-Hz frequency modulated or warble tone. Hong and Santosa^16^ performed a similar experiment to study hemodynamic responses for “natural” sound stimuli, such as English and non-English words, annoying sounds, and nature sounds. Issa et al.^18^ measured the hemodynamic changes in the auditory cortex when presented with a pure tone stimulus of 750 and 8000 Hz as well as broadband noise. The primary goal of these experiments was to measure or image focal, i.e., localized, changes in cerebral tissue oxygenation within the auditory cortex—this could be thought of as the fundamental question of fNIRS experiments. The primary auditory cortex in humans spans $∼1650\;{\mathrm{mm}}^{3}$ within Heschl’s gyrus of the temporal lobe and is organized along multiple functional dimensions, the most prominent one being tonotopic.^19^^,^^20^ We, therefore, expect that pure tone stimuli will activate a more focal region of the auditory cortex, whereas broadband noise will activate a broader region.^19^^,^^21^^,^^22^

Traditionally, fNIRS has been implemented using a continuous wave approach [continuous wave near-infrared spectroscopy (CW-NIRS)] because it offers a fast and convenient way to measure focal changes in oxygenated hemoglobin ($\Delta C_{\mathrm{HbO}}$) and deoxygenated hemoglobin ($\Delta C_{\mathrm{HbR}}$). Indeed, most commercial implementations of NIRS follow this approach. In CW-NIRS, tissue is illuminated with a light source of constant intensity, and the attenuated light emitted from the surface of tissue, measured 1 to 2.5 cm away on the tissue surface, is used to calculate the absorbance or optical density. Changes in the optical density, e.g., due to neuronal activation, are converted to relative changes in the concentration of tissue chromophores using the modified Beer–Lambert (MBL) law.^1^ The MBL approach implements a simple correction factor (differential path length factor) to account for the extension of light pathlength due to multiple scattering in tissue.^23^ A more quantitative alternative to CW-NIRS is frequency domain NIRS (FD-NIRS), which estimates absolute concentrations of oxygenated and deoxygenated hemoglobin from measurements that can be directly fit to solutions to the photon diffusion equation.^24^ In its most common implementation, FD-NIRS measures the intensity and phase shift of backscattered light from multiple source detection separations, when tissue is illuminated by an intensity modulated source at different wavelengths. The intensity modulated source sets up a diffusive photon density wave in tissue, the wave vector of which is used to estimate optical tissue properties. Since this approach directly estimates scattering and absorption coefficients of tissue, it holds the potential to measure baseline concentrations of oxygenated and deoxygenated hemoglobin. However, despite being a more quantitative alternative and the availability of commercial FD-NIRS instruments (e.g., OxiplexTS, Imagent from ISS Inc.), studies that use FD-NIRS have primarily focused on global changes in concentrations of $C_{\mathrm{HbO}}$ and $C_{\mathrm{HbR}}$ such as hypoxia and cerebral ischemia,^15^^,^^25^^–^^28^ and the literature that discusses the implementation of this more quantitative technique to measure the focal changes in $C_{\mathrm{HbO}}$ and $C_{\mathrm{HbR}}$ such as those involving functional activation experiments is scant.

In this study, we explored this apparent discrepancy and compared typical implementations of CW- and FD-NIRS instruments to measure functional activation. Specifically, we characterized the hemodynamic response of the human auditory cortex due to functional activation using a commercial FD-NIRS instrument and analyzed the results using both CW and multi-distance FD approaches. Using *in vivo* data from functional activation of healthy human auditory cortex, we demonstrate that hemodynamic responses estimated from CW- and multi-distance FD-NIRS approaches differ significantly even though they are measured at the same time and from the same physical position. Furthermore, using finite-element simulations (NIRFAST^29^), we suggest that FD-NIRS estimates of cerebral oxygenation measured using multi-distance methods tend to overestimate focal hemodynamic changes, in part due to the partial volume effect, when compared with single-distance CW approaches.

## 2 Experimental Methods

### 2.1 Frequency Domain Near-Infrared Spectroscopy Instrument

Cerebral hemodynamic changes due to functional activation were measured using a commercial frequency-domain near-infrared spectroscopy instrument (Imagent, ISS Inc., IL, USA). The instrument features eight laser diodes as light sources at two different wavelengths (690 and 830 nm) and eight photomultiplier tubes (A through H) for use as detectors. The intensity of the lasers was modulated at 110 MHz. The instrument demodulates the photon fluence measured at each detector to estimate AC, DC, and phase of the detected photon density wave.^30^ The laser diodes were time multiplexed to illuminate at one source position/wavelength (1 through 4) at a time, whereas photon fluence is measured at all detectors simultaneously, resulting in an effective total frame rate of 10 Hz. The system was warmed up for 10 min prior to the start of the measurement. Light from the sources was directed to source positions in a custom optical probe (see Sec. 2.2) using a bifurcated fiber-optic cable (multi-mode bundle, 1-mm diameter). Light detected from eight detector positions was directed to photomultiplier tubes A through H using 3-mm-diameter fiber bundles. Thus AC, DC, and phase measurements at each detector facilitated multi-distance frequency domain NIRS estimations of baseline tissue optical properties. The average source–detector separation was ∼3  cm. Table 1 lists the source–detector separations (in cm) for each detection and source position.

**Table 1:** Distance (cm) between the sources and detectors for fiberoptic probe shown in Fig. 1.

	Source 1	Source 2	Source 3	Source 4
Detector A	2.2	2.9	3.8	4.6
Detector B	1.6	2.1	2.9	3.6
Detector C	3.6	2.9	2.1	1.6
Detector D	4.6	3.8	2.9	2.2
Detector E	2.2	2.9	3.8	4.6
Detector F	1.6	2.1	2.9	3.6
Detector G	3.6	2.9	2.1	1.6
Detector H	4.6	3.8	2.9	2.2

### 2.2 Probe Design and Placement

**Fig. 1:**
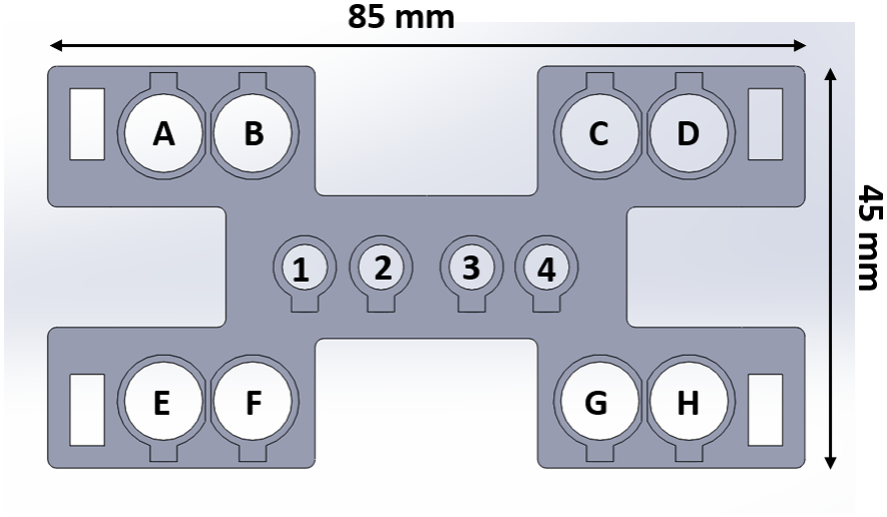
Schematic of custom fiberoptic probe with four source positions and eight detector positions. Source positions are denoted 1 through 4, and detector positions are denoted A to H. The corresponding source–detector separations are listed in Table 1.

**Fig. 2:**
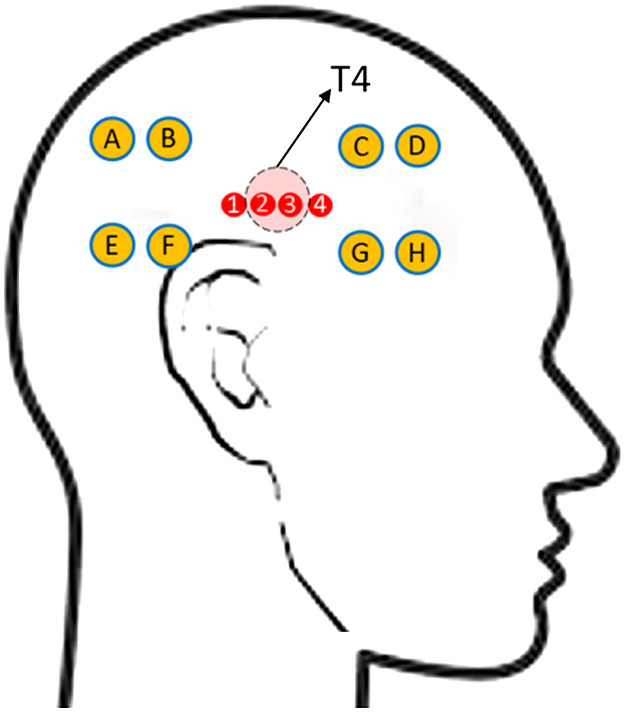
Schematic representing positioning of sources and detectors on the scalp over the auditory cortex. Red circles represent the source positions, the center of which is placed above the T4 position over the right ear. Yellow circles represent detector positons, with detectors A, B, E, and F sampling posterior auditory cortex and detectors C, D, G, and H sampling the anterior auditory cortex.

A custom optical probe was 3D-printed to hold source/detector fibers at their required separations. A schematic for the arrangement for source and detector fibers (four source positions and eight detector positions) is shown in Fig. 1. Large circles highlight the locations of the detector fibers, and the smaller circles highlight the locations of the source fibers. Using the international 10-20 system as a guide, the probe was placed such that center of the sources was on either side of the T4 position or over the right auditory cortex^7^^,^^16^^,^^18^^,^^31^^,^^32^ as shown in Fig. 2. The T4 position was identified on each subject from individual measurements of head circumference, inion–nasion, and tragus–tragus distances. The probe was held in place using two thin Velcro straps on both sides. A USB otoscope was used to minimize the effect of hair at the source and detector positions after the placement of the probe. The scope was used to check and move hair from obstructing the fibers. The intensity values were checked after the placement of the probe to ensure a good signal response.

### 2.3 Subjects

Nine healthy adults (six female and three male), median age of 29 years (range: 20 to 35 years old), with normal hearing (≤25  dB HL, 250 to 8000 Hz), participated in this study. All procedures were approved by USF’s Institutional Review Board, and subjects provided written informed consent prior to participation.

### 2.4 Experimental Protocol

Functional activation measurements were performed in a double-walled, sound-treated booth with lights dimmed to limit the effect of ambient lights on the measurements. Responses to two different auditory stimuli were evaluated, 1000 Hz pure tone and broadband noise; the order of presentation was randomized across subjects. The stimuli were generated digitally [RP2, Tucker Davis Technologies (TDT), Alachua, FL], routed to a programmable attenuator (PA5, TDT), headphone buffer (HB6, TDT), and presented binaurally at 70 dB SPL via insert earphones (ER-2, Etymotic Research Inc., Elk Grove, IL). Stimuli were controlled using SigGen and BioSig software (TDT) with each stimulus having a duration of 10 s, a 30-s interstimulus interval, and 21 repetitions. The experiment protocol is similar to those in publications that have used fNIRS to study the auditory response.^16^^,^^33^^,^^34^ The overall recording time was ∼14  min including a 60 s baseline period prior to stimuli presentation. NIRS measurements of average intensity (DC), amplitude (AC), and phase (ϕ) were continuously recorded along with trigger markers to indicate the start and end of the stimulus presentation. Data were postprocessed to estimate hemodynamic changes due to functional activation.

### 2.5 CW-NIRS Analysis

The MBL approach was used to calculate relative changes in concentrations of oxygenated hemoglobin ($C_{\mathrm{HbO}}$) and deoxygenated hemoglobin ($C_{\mathrm{HbR}}$) due to functional activation, with custom MATLAB scripts (Mathworks, Natick, MA). To reduce the impact of the room or other stray lights on the recorded data, we used the AC amplitude to perform the CW-NIRS analysis.^35^ The recorded amplitude at each source–detector separation was first converted to optical density by normalizing the intensity time traces at each wavelength with the corresponding average intensity at baseline. A moving average window (0.4 s) was used to remove motion artifacts in the data;^31^ the local variance in OD within the moving window was calculated, and regions/trials that exhibited large local standard deviation (>2 times mean variance) were excluded from the analysis. Motion artifact corrected time courses were band pass filtered (0.01 to 0.5 Hz) and split into 30 s epochs corresponding to each trial of auditory stimuli. For each trial, the change in optical density was computed by subtracting the baseline (i.e., prestimulation) optical density from the measured optical density. Finally, changes in optical density were then converted to changes in $C_{\mathrm{HbO}}$ and $C_{\mathrm{HbR}}$ on a trial-by-trial basis by inverting the following equation:

$$[\begin{array}{c} \Delta \mathrm{OD}(\lambda_{1})\\ \Delta \mathrm{OD}(\lambda_{2}) \end{array}]=\;[\begin{array}{cc} \epsilon_{\mathrm{Hbo}}(\lambda_{1})\;\mathrm{DPF}(\lambda_{1})\rho & \epsilon_{\mathrm{HbR}}(\lambda_{1})\mathrm{DPF}(\lambda_{1})\rho \\ \epsilon_{\mathrm{Hbo}}(\lambda_{2})\mathrm{DPF}(\lambda_{2})\rho & \epsilon_{\mathrm{HbR}}(\lambda_{2})\mathrm{DPF}(\lambda_{2})\rho \end{array}][\begin{array}{c} \Delta C_{\mathrm{HbO}}(\lambda_{1})\\ \Delta C_{\mathrm{HbR}}(\lambda_{2}) \end{array}].$$ (1)

Here DPF is the differential pathlength factor; DPF is detector specific for each subject and was calculated as $\mathrm{DPF}(\lambda )=\frac{\sqrt{3\mu_{s}^{\prime }(\lambda )}}{2\sqrt{\mu_{a}(\lambda )}}$, where $\mu_{a}$ and $\mu_{s}^{\prime }$ were estimated from the baseline data calculated with FD-NIRS analysis,^27^ ρ is the source–detector separation in cm, and $\epsilon_{\mathrm{HbO}/\mathrm{HbR}}(\lambda_{i})$ is the molar extinction coefficient of HbO/HbR at wavelength $\lambda_{i}$ in ${\mathrm{cm}}^{-1}\;{\mathrm{mol}}^{-1}$.^36^^,^^37^ CW-NIRS data are especially susceptible to instrumental drifts and coupling errors. Hence, the hemodynamic responses were detrended to remove gradual drifts in the data. Detrending was performed on a trial-by-trial basis to reduce the effects of any long-term data drifts. Results from multiple trials and from all subjects were averaged. Figures S1 and S2 in the Supplementary Material show the CW-NIRS data processing pipeline for an exemplar subject.

### 2.6 Multi-Distance Frequency Domain NIRS Analysis

Estimates of absolute concentrations of oxygenated and deoxygenated hemoglobin were computed for the functional activation measurements using the multi-distance frequency domain approach.^30^^,^^38^ Intensity and phase data were corrected for motion artifacts, as described earlier. The corrected data were filtered using a low-pass filter with a cutoff frequency of 0.1 Hz. A phantom calibration procedure was employed to estimate and correct for probe-tissue coupling coefficients.^39^^,^^40^ At each time point, calibrated and filtered amplitudes (A) measured at each detector were linearized ($\log(\rho^{2}A(\rho ))$) and plotted as a function of source–detector separation (ρ); a linear fit was performed to estimate the slope $k_{r}$. Similarly, a linear fit to phase (ϕ) as a function of ρ was performed to estimate the slope $k_{i}$. Tissue optical properties $\mu_{a}$ and $\mu_{s}^{\prime }$ were estimated from the following expressions:^40^

$$\mu_{a}(\lambda )=\frac{\omega }{2v}(\frac{\kappa_{r}(\lambda )}{\kappa_{i}(\lambda )}-\frac{\kappa_{i}(\lambda )}{\kappa_{r}(\lambda )}),\;\mu_{s}^{\prime }(\lambda )=\frac{2v}{3\omega }(-\kappa_{r}(\lambda )\kappa_{i}(\lambda )),$$

where $\omega =2\pi f_{\mathrm{mod}}$, $f_{\mathrm{mod}}$ is the modulation frequency and v is the speed of light in the tissue. The measured absorption coefficient was corrected for water absorption ($\mu_{a}^{830}=0.029\;{\mathrm{cm}}^{-1}$ and $\mu_{a}^{690}=0.0049\;{\mathrm{cm}}^{-1}$), assuming that water accounts for 75% of brain matter,^41^^,^^42^^–^^44^ and converted to time courses of concentrations of oxygenated ($C_{\mathrm{HbO}}$) and deoxygenated hemoglobin ($C_{\mathrm{HbR}}$). In a manner similar to CW-NIRS analysis, the time courses of $C_{\mathrm{HbO}}$ and $C_{\mathrm{HbR}}$ were split into epochs corresponding to auditory stimuli. Changes in concentrations of $C_{\mathrm{HbO}}$ and $C_{\mathrm{HbR}}$ were computed by subtracting the baseline (i.e., prestimulus) concentration from the respective time courses of $C_{\mathrm{HbO}}$ and $C_{\mathrm{HbR}}$. Results from multiple trials and from all subjects were averaged. Note that some detectors feature FD-NIRS measurements at a source–detector separation of 1.6 cm. Since extracerebral contributions are likely to be significant at these separations, amplitude and phase data collected at this short separation were excluded from FD-NIRS analysis. Figures S3 and S4 in the Supplementary Material show the FD-NIRS data processing pipeline for an exemplar subject.

### 2.7 Statistical Analysis of Functional Activation Responses

The average changes in oxygenated and deoxygenated hemoglobin during the activation period were calculated to quantitatively characterize the functional activation responses. Briefly, the highest point (i.e., maximum change) in the hemodynamic response within the activation period was identified. The average change in concentrations 1.5 s before and after the time of maximum response was computed and used as a quantitative metric of functional activation. The Wilcoxon signed rank test was performed to test if the average hemodynamic change was statistically greater than zero. In addition, the subject-wise paired Wilcoxon signed rank test was performed to test if the CW-NIRS responses at each source–detector separation were smaller from the corresponding FD-NIRS responses. All statistical testing was performed at 5% significance level. The statistical analyses performed in this paper are focused on the oxygenated hemoglobin because signals from the cortex are influenced by oxygenated hemoglobin changes to a greater extent (76%) and oxygenated hemoglobin responses were more robust to noise.^45^^,^^46^

## 3 Results

### 3.1 CW-NIRS and FD-NIRS Estimates of Hemodynamic Changes due to Functional Activation

We first highlight the baseline concentrations of oxygenated and deoxygenated hemoglobin at each detector for all subjects (Table 2). Baseline concentrations were obtained by multi-distance FD-NIRS analysis of phase and amplitude. Note that the source–detector separations (Table 1) vary for each detector (per probe geometry in Fig. 1). Therefore, the results in Table 2 represent an ensemble sampling of the auditory cortex regions both laterally and in depth. FD-NIRS analysis was possible on data from only six subjects. One subject’s data was unusable due to errors during the calibration process. Data from two other subjects were excluded because intermittent saturation effects during the experiment rendered the data unusable. CW-NIRS analysis utilizes the DPF calculated with FD-NIRS analysis; hence the data from six subjects were used.

**Table 2:** Baseline concentrations of oxygenated and deoxygenated hemoglobin (μM) averaged across all subjects for detectors A to H.

	$C_{\mathrm{HbO}}$ (μM)	$C_{\mathrm{HbR}}$ (μM)
Detector A	70.7 ± 11.7	26.8 ± 4.07
Detector B	69.0 ± 13.4	33.1 ± 8.9
Detector C	42.0 ± 16.2	17.9 ± 3.1
Detector D	48.0 ± 12.6	20.5 ± 3.2
Detector E	58.9 ± 10.2	26.7 ± 4.5
Detector F	58.8 ± 3.2	28.1 ± 2.5
Detector G	39.5 ± 13.5	20.5 ± 3.5
Detector H	41.3 ± 13.1	20.1 ± 2.5

**Fig. 3:**
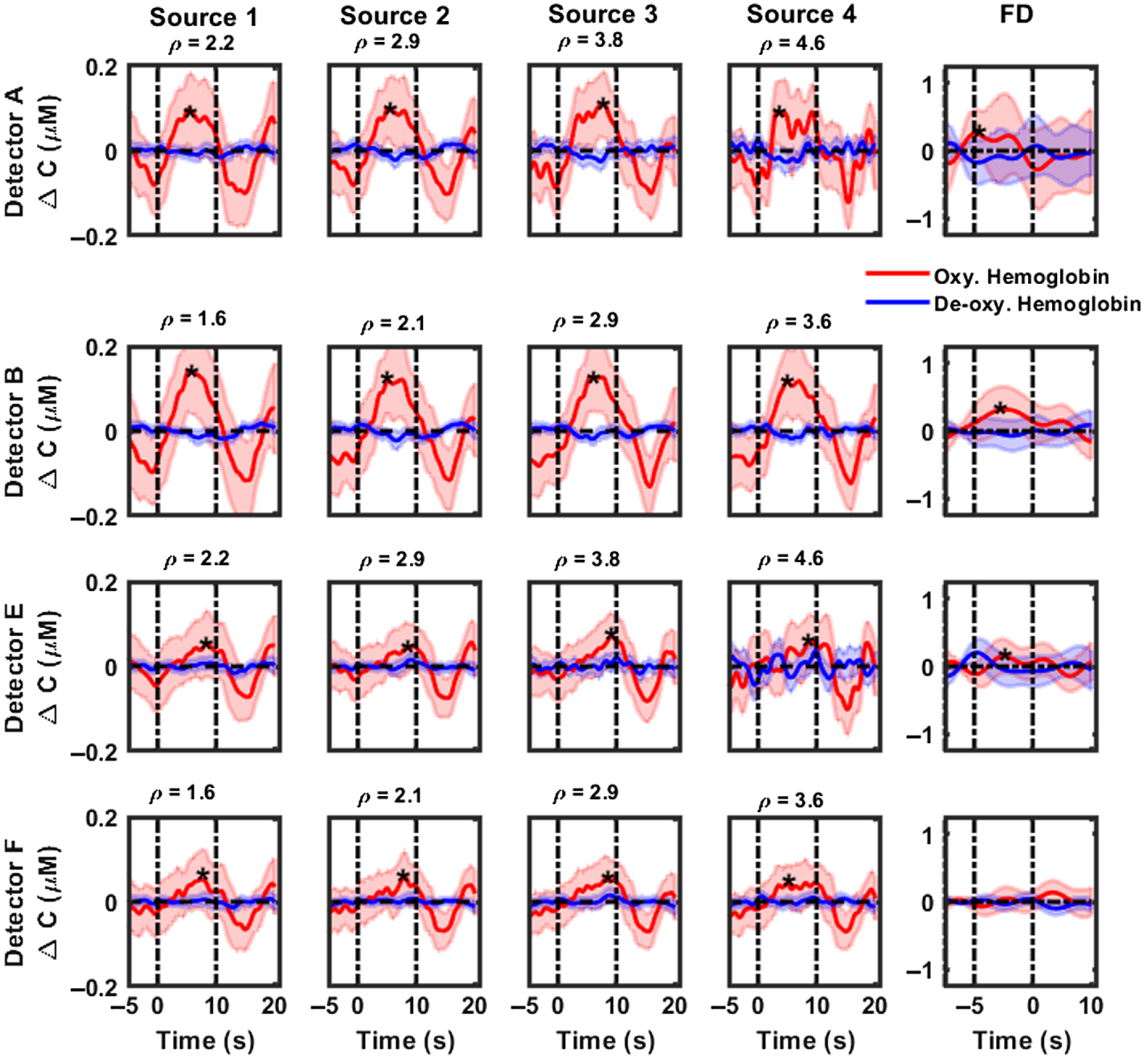
CW-NIRS and FD-NIRS analyses of functional activation (1000 Hz pure tone) recorded over the posterior auditory cortex (detectors A, B, E, and F) from all subjects. Rows represent hemodynamic responses recorded at each detector. The first four columns highlight responses recoded at each source position using CW-NIRS (left to right, increasing source–detector separation) and FD-NIRS (last column). In each plot, changes in concentration of oxygenated hemoglobin are plotted in red, and the concentration of deoxyhemoglobin is plotted in blue. The title of each subplot denotes the source–detector separation in cm for that measurement channel. Note that the y axis scale for FD-NIRS measurements is larger than that of CW-NIRS measurements. Dashed vertical lines indicate the start and end of the stimulus. Shaded regions indicate the variance in the responses. Note that source 1 is closest to and source 4 is farthest from all detectors. Plots marked with a “*” indicate statistically significant responses.

**Fig. 4:**
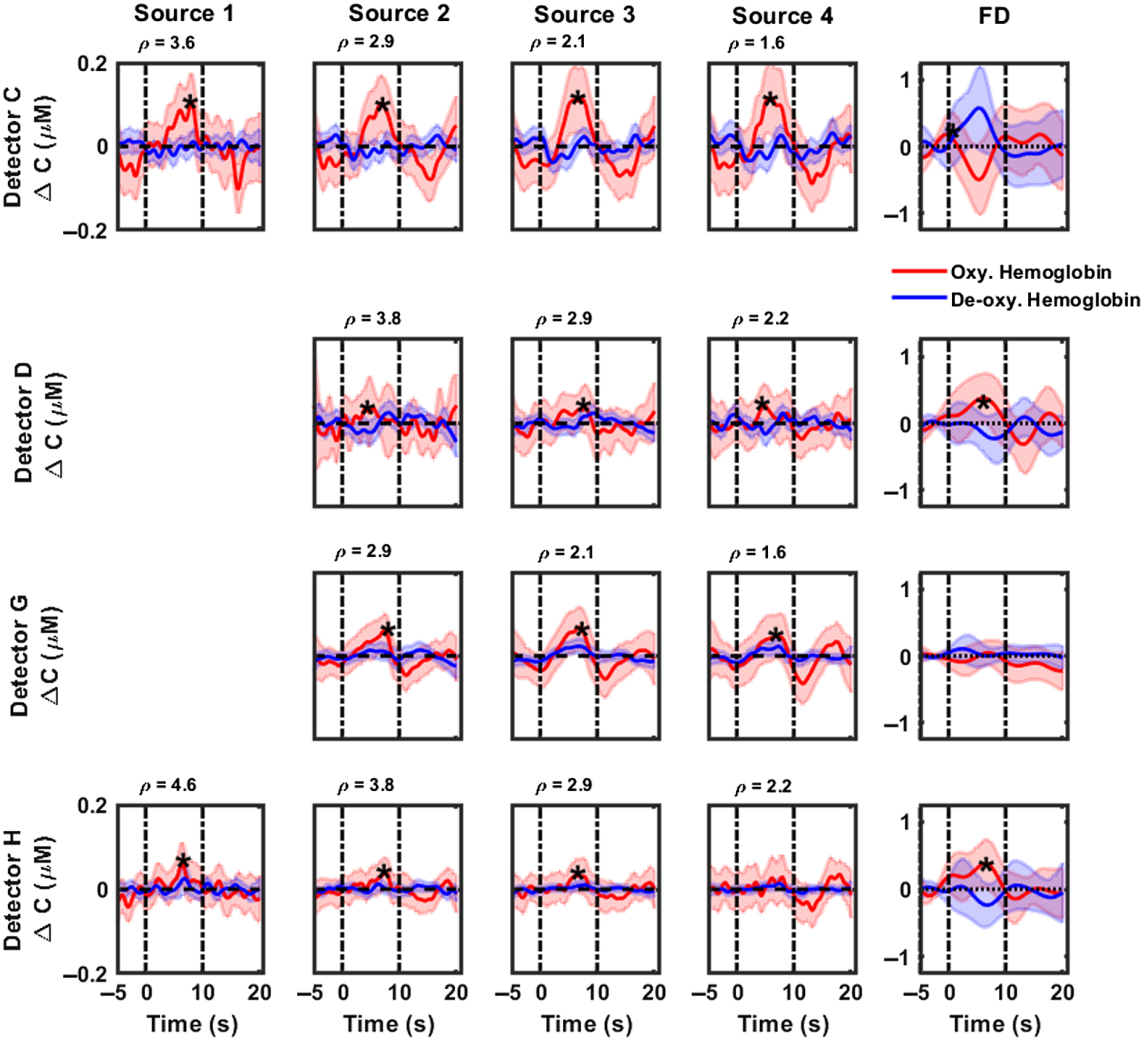
CW-NIRS and FD-NIRS analyses of functional activation (1000 Hz pure tone) recorded over the anterior auditory cortex (detectors C, D, G, and H) from all subjects. Rows represent hemodynamic responses recorded at each detector. The first four columns highlight responses recoded at each source position using CW-NIRS (left to right, decreasing source–detector separation) and FD-NIRS (last column). In each plot, changes in concentration of oxygenated hemoglobin are plotted in red, and the concentration of deoxyhemoglobin is plotted in blue. The title of each subplot denotes the source–detector separation in cm for that measurement channel. Note that the y axis scale for FD-NIRS measurements is larger than that of CW-NIRS measurements. Dashed vertical lines indicate the start and end of the stimulus. Shaded regions indicate the variance in the responses. Note that source 4 is closest to and source 1 is farthest from all detectors. Plots for source 1. Detector D and G are not included here because they were too noisy. Plots marked with a “*” indicate statistically significant responses.

Figures 3 and 4 highlight the cumulative hemodynamic response curves from CW-NIRS (first four columns) and FD-NIRS (last column) analyses of 1000 Hz pure tone activation, recorded over the posterior and anterior auditory cortices respectively, from six subjects. Changes in concentrations of oxygenated hemoglobin are displayed as solid red lines, with corresponding shaded regions denoting 95% confidence intervals. Similarly, changes in concentrations of deoxygenated hemoglobin and corresponding 95% confidence intervals are highlighted in blue. The period between the dashed vertical lines, i.e., between 0 and 10 s, represents the activation duration. With CW-NIRS analysis, a robust increase of 0.107 to 0.13  μM is observed in oxygenated hemoglobin (maximum value within duration of stimulus presentation), primarily in the auditory cortex region superior to the T4 position (detectors A, B, and C). Hemodynamic responses were quantified as described in Sec. 2.7 (see also Table 3). Statistically significant responses (Wilcoxon signed rank test, testing for response >0) were observed in most source–detector pairs in the posterior auditory cortex (marked by a “*” in Fig. 3). Hemodynamic changes measured in the anterior auditory cortex (Fig. 4) were modest compared with those in the posterior auditory cortex, indicating the presence of the tonotopic response to pure tone stimuli. No significant changes were observed in the concentration of deoxygenated hemoglobin. We also note that the responses become noisier for detectors A and E as the source–detector separation increases. Detectors B and F show a decrease in activation with increases in the source–detector separation. The results of FD-NIRS analysis are highlighted in the last columns in Figs. 3 and 4. Note that the y axis scale for these plots spans a larger range than for CW-NIRS. In general, FD-NIRS analysis yielded hemodynamic changes that were greater than those for CW-NIRS. The strongest (maximum) response was quantified to be 0.31  μM in detector B. Hemodynamic responses were quantified as described in Sec. 2.7 (see also Table 3); statistically significant (Wilcoxon signed rank test, testing for response >0) responses are marked with a “*.”

**Table 3:** Comparison of change in concentration of oxygenated hemoglobin ($\Delta C_{\mathrm{HbO}}$) due to auditory stimulation (1000 Hz pure tone), estimated with CW-NIRS (columns 2 to 5) and FD-NIRS (column 6) analysis methods. Magnitude of response was quantified for each subject using methods in Sec. 2.7. Average and standard deviation of the responses across different subjects are displayed here.

Detectors	$\Delta C_{\mathrm{HbO}}$ in μM for 1000 Hz pure tone				
	Source 1	Source 2	Source 3	Source 4	FD-NIRS
A	0.08 ± 0.01	0.1 ± 0.01	0.1 ± 0.01	0.06 ± 0.02^a^	0.24 ± 0.03
B	0.13 ± 0.02	0.11 ± 0.02	0.12 ± 0.01	0.11 ± 0.01	0.28 ± 0.06
C	0.09 ± 0.02	0.11 ± 0.02	0.12 ± 0.02	0.11 ± 0.02	0.14 ± 0.03
D	—	0.03 ± 0.01	0.04 ± 0.01	0.01 ± 0.01	0.36 ± 0.02
E	0.04 ± 0.006^a^	0.04 ± 0.01^a^	0.06 ± 0.01^a^	0.06 ± 0.01^a^	0.15 ± 0.02
F	0.04 ± 0.01	0.04 ± 0.01^a^	0.05 ± 0.005^a^	0.04 ± 0.005^a^	0.04 ± 0.01
G	—	0.04 ± 0.02^a^	0.06 ± 0.01	0.05 ± 0.02	0.03 ± 0.02
H	0.03 ± 0.01^a^	0.02 ± 0.01	0.03 ± 0.01	0.01 ± 0.01	0.4 ± 0.03

a The source–detector pairs with statistically significant difference between CW-NIRS and FD-NIRS.

**Table 4:** Comparison of change in concentration of oxygenated hemoglobin ($\Delta C_{\mathrm{HbO}}$) due to auditory stimulation (70 dB SPL broadband noise), estimated with CW-NIRS (columns 2 to 5) and FD-NIRS (column 6) analysis methods. Magnitude of response was quantified for each subject using methods in Sec. 2.7. Average and standard deviation of the responses across different subjects are displayed here.

Detectors	$\Delta C_{\mathrm{HbO}}$ in μM for broadband noise				
	Source 1	Source 2	Source 3	Source 4	FD-NIRS
A	0.04 ± 0.01^a^	0.07 ± 0.01^a^	0.09 ± 0.02^a^	0.07 ± 0.02^a^	0.38 ± 0.03
B	0.07 ± 0.01	0.09 ± 0.01	0.12 ± 0.01	0.12 ± 0.01	0.15 ± 0.01
C	0.1 ± 0.01	0.1 ± 0.01	0.13 ± 0.01	0.11 ± 0.01	0.33 ± 0.02
D	—	0.05 ± 0.01	0.04 ± 0.01	0.03 ± 0.008	0.14 ± 0.01
E	0.05 ± 0.01	0.05 ± 0.01^a^	0.05 ± 0.01^a^	0.06 ± 0.01^a^	0.17 ± 0.03^a^
F	0.06 ± 0.01^a^	0.05 ± 0.01^a^	0.06 ± 0.01^a^	0.06 ± 0.01^a^	0.1 ± 0.02
G	—	0.04 ± 0.005	0.04 ± 0.01	0.05 ± 0.01	0.027 ± 0.03
H	0.04 ± 0.01	0.05 ± 0.01	0.05 ± 0.01	0.06 ± 0.02^a^	0.023 ± 0.01

a The source–detector pairs with statistically significant difference between CW-NIRS and FD-NIRS.

**Fig. 5:**
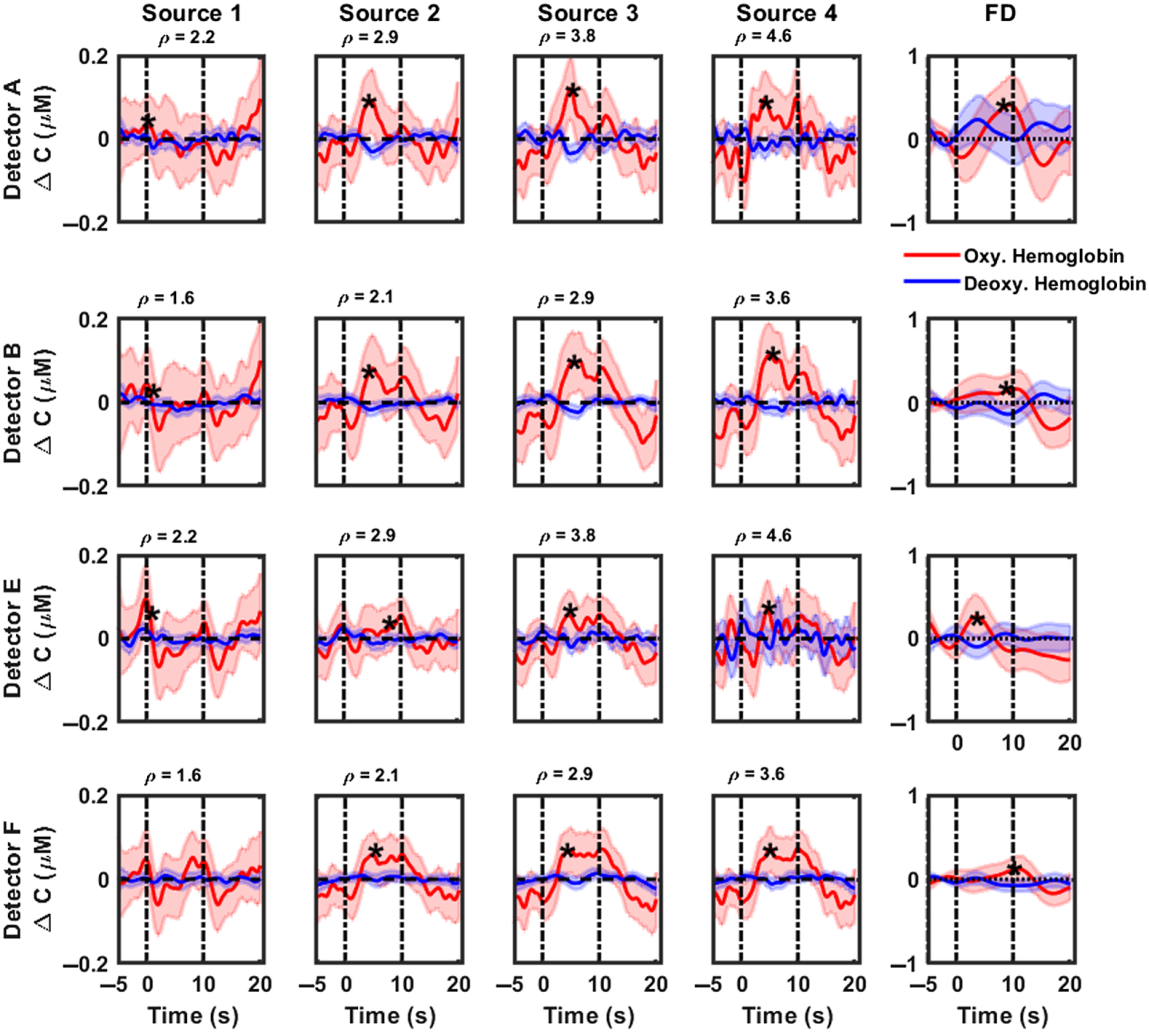
CW-NIRS and FD-NIRS analyses of functional activation (70 dB SPL broadband noise) recorded over the posterior auditory cortex (detectors A, B, E, and F) from all subjects. Rows represent hemodynamic responses recorded at each detector. The first four columns highlight responses recoded at each source position using CW-NIRS (left to right, increasing source–detector separation) and FD-NIRS (last column). In each plot, changes in concentration of oxygenated hemoglobin are plotted in red, and the concentration of deoxyhemoglobin is plotted in blue. The title of each subplot denotes the source–detector separation in cm for that measurement channel. Note that the y axis scale for FD-NIRS measurements is larger than that of CW-NIRS measurements. Dashed vertical lines indicate the start and end of the stimulus. Shaded regions indicate the variance in the responses. Note that source 1 is closest to and source 4 is farthest from all detectors. Plots marked with a “*” indicate statistically significant responses.

**Fig. 6:**
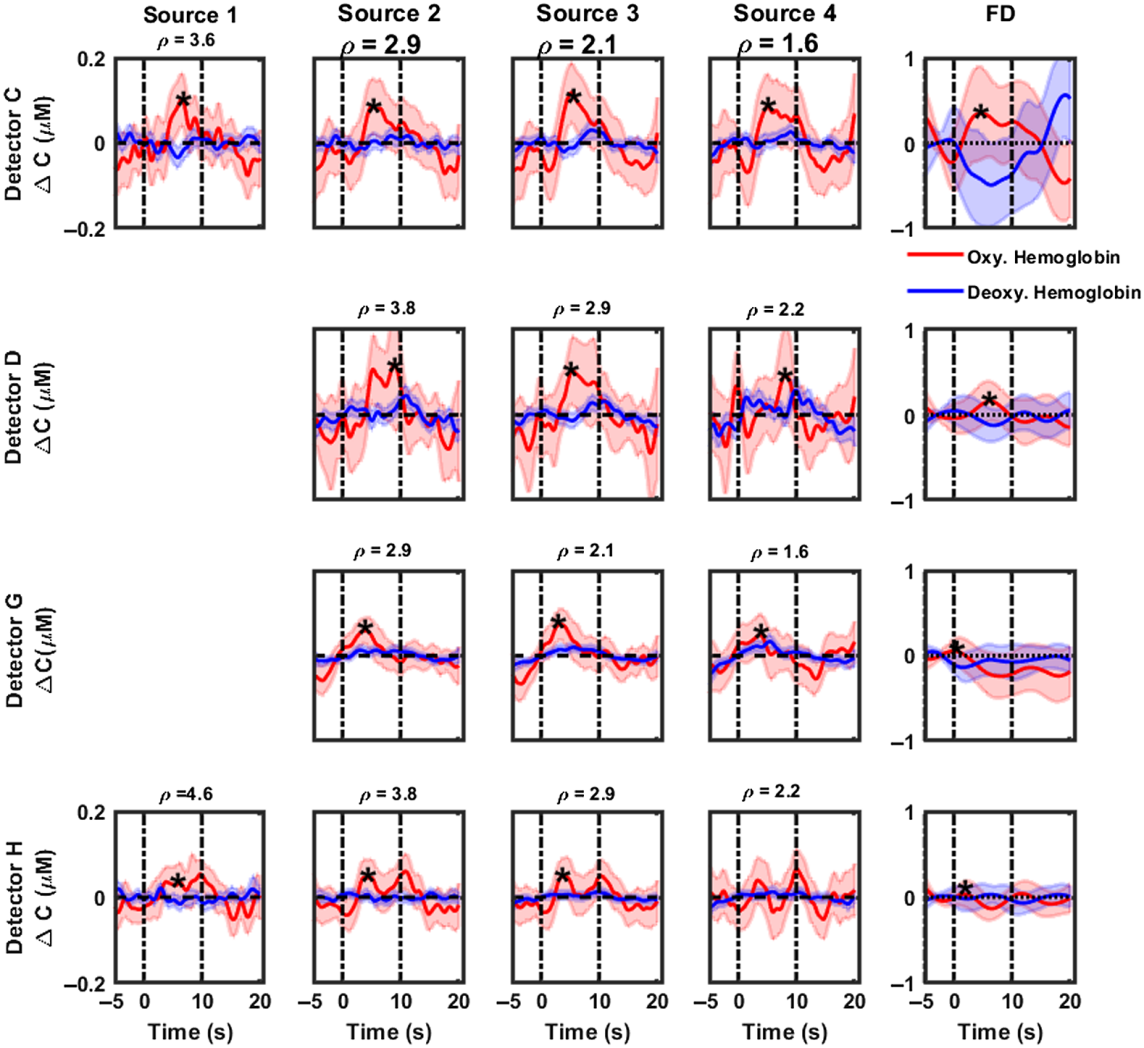
CW-NIRS and FD-NIRS analyses of functional activation (70 dB SPL broadband noise) recorded over the anterior auditory cortex (detectors C, D, G, and H) from all subjects. Rows represent hemodynamic responses recorded at each detector. The first four columns highlight responses recoded at each source position using CW-NIRS (left to right, decreasing source–detector separation) and FD-NIRS (last column). In each plot, changes in concentration of oxygenated hemoglobin are plotted in red, and the concentration of deoxyhemoglobin is plotted in blue. The title of each subplot denotes the source–detector separation in cm for that measurement channel. Note that the y axis scale for FD-NIRS measurements is larger than that of CW-NIRS measurements. Dashed vertical lines indicate the start and end of the stimulus. Shaded regions indicate the variance in the responses. Note that source 4 is closest to and source 1 is farthest from all detectors. Detector D and G are not included here because they were too noisy. Plots marked with a “*” indicate statistically significant responses.

Figures 5 and 6 display the cumulative hemodynamic response curves from CW-NIRS (first four columns) and FD-NIRS (last column) analyses of 70 dB SPL broadband noise activation, recorded over the posterior and anterior auditory cortices, respectively, from all subjects. Changes in concentrations of oxygenated/deoxygenated hemoglobin are displayed as solid red/blue lines, with corresponding shaded regions denoting 95% confidence intervals. The period between the dashed vertical lines, i.e., between 0 and 10 s, represents the activation duration. On average, the hemodynamic responses to broadband noise activation were weaker compared with those of pure tone, more diffuse in nature, and stronger in the anterior auditory cortex. This is in line with the tonotopic organization of the auditory cortex.^19^^,^^21^^,^^22^ With CW-NIRS analysis, the strongest response (maximum value within duration of stimulus presentation) was observed in detectors B and C (0.12 and 0.13  μM, respectively). Hemodynamic responses were quantified as described in Sec. 2.7 (see also Table 4). Statistically significant responses (Wilcoxon signed rank test, testing for response >0) are marked with a “*” in Figs. 5 and 6. The results of FD-NIRS analysis are highlighted in the last columns in Figs. 5 and 6. Note that the y axis scale for these plots spans a larger range than with CW-NIRS. As with pure tone, FD-NIRS analysis of broadband noise stimulus yielded hemodynamic changes that were greater than those of CW-NIRS. Hemodynamic responses were quantified as described in Sec. 2.7 (see also Table 4); statistically significant (Wilcoxon signed rank test, testing for response >0) are marked with a “*.”

### 3.2 Comparison of FD-NIRS and CW-NIRS Hemodynamic Responses to Functional Activation

Figures 3–6 show that hemodynamic responses to auditory stimulation measured using FD are generally larger than the corresponding CW-NIRS responses. Although both approaches are consistent in identifying the primary activation region, the magnitude of responses is significantly different. Notably, the results from the FD-NIRS analysis show a large range (0.35 to 0.41  μM) for the changes in concentration of HbO, whereas in the MBL analysis approach, we observed similar changes but over a smaller range (0.05 to 0.16  μM). We quantified the hemodynamic responses for each detector (FD-NIRS) and each source–detector pair (CW-NIRS) using the methods describes in Sec. 2.7—these are summarized in Table 3 (pure tone stimulus) and Table 4 (broadband noise). On average, FD-NIRS responses were greater than CW-NIRS responses by a factor of 2. To evaluate if the hemodynamic responses from FD-NIRS were greater than those of CW-NIRS, subject-wise paired Wilcoxon sign rank tests were performed. Briefly, the hemodynamic responses recorded at each detector (FD-NIRS analysis) were compared with the corresponding responses for all source–detector separations (CW-NIRS), paired subject-wise. For pure tone, source 4 in detector A (ρ=4.6  cm), sources 1 to 4 in detector E (ρ=2.2 to 4.6 cm), sources 2 to 4 in detector F (ρ=2.1 to 3.6 cm), source 2 in detector G (ρ=2.9), and source 1 in detector H (ρ=4.6  cm) were found to be statistically significant. Most of these source–detector pairs correspond to detectors in the posterior auditory cortex. For broadband noise, all sources in detector A (ρ=2.2 to 4.6 cm), sources 2 to 4 in detector E (ρ=2.9 to 4.6 cm), all sources in detector F (ρ=1.6 to 3.6 cm), and source 4 in detector H (ρ=2.2  cm) were found to be statistically significant.

### 3.3 Numerical Simulation of FD-NIRS and CW-NIRS Hemodynamic Responses to Functional Activation

**Fig. 7:**
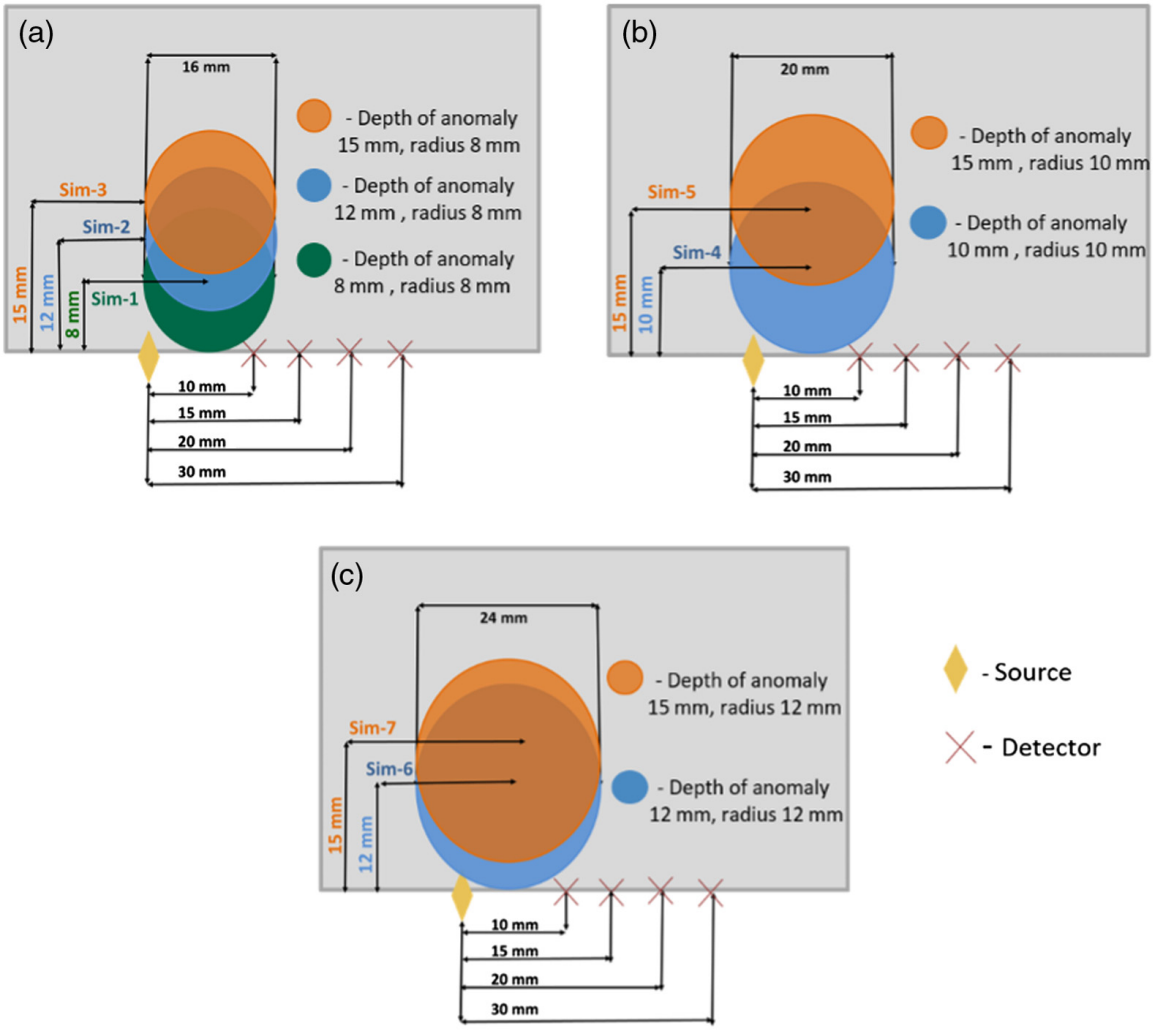
Schematic of homogenous slab geometry used for finite-element (NIRFAST) simulations. Yellow rhombus indicates of the position of the detector, and crosses indicate the positions of four sources, with source–detector separations ranging from 1 to 3 cm. The colored circles highlight the position of circular anomalies that simulates the region with functional activation. The background properties for tissues were $C_{\mathrm{HbO}}=0.01\;\mathrm{mM}$, $C_{\mathrm{HbR}}=0.01\;\mathrm{mM}$, scattering amplitude = 1, and scattering power = 1. The optical properties of the anomaly were $C_{\mathrm{HbO}}=0.06\;\mathrm{mM}$, $C_{\mathrm{HbR}}=0.005\;\mathrm{mM}$, scattering amplitude = 1.34, and scattering power = 0.56.

To validate our results, we used a finite-element-based forward model of tissue light propagation, implemented in NIRFAST,^29^ to simulate functional activation responses that could be analyzed using both CW-NIRS and FD-NIRS approaches. Briefly, a homogenous tissue surface was configured as a 50  mm×80  mm 2D mesh (over 16k nodes, 0.5 mm node distance) with optical properties of $C_{\mathrm{HbO}}=0.01\;\mathrm{mM}$, $C_{\mathrm{HbR}}=0.01\;\mathrm{mM}$, and both scattering amplitude and scattering power equal to 1. In seven different simulations, circular anomalies (simulating focal activation) of different radii (8, 10, and 12 mm) were placed at multiple depths below the tissue surface (15, 10, and 8 mm) as shown in Fig. 7. The size and depths of the anomaly were selected to mimic activation in different sections of the auditory cortex.

Optical properties of the anomaly were set to of $C_{\mathrm{HbO}}=0.06\;\mathrm{mM}$, $C_{\mathrm{HbR}}=0.005\;\mathrm{mM}$,^7^^,^^32^^,^^34^^,^^41^ scattering amplitude of 1.34, and scattering power of 0.56. NIRFAST was used to solve the forward model of frequency domain photon migration (100 MHz modulation frequency, wavelengths of 690 and 830 nm) to record the amplitude and phase of the photon fluence rate at source–detector separations of 10, 15, 20, and 30 mm. Simulated amplitude and phase measurements were analyzed using the FD-NIRS approach, whereas amplitude was analyzed with the CW-NIRS approach to estimate the change (from baseline) in concentration of oxygenated and deoxygenated hemoglobin. The % error in hemodynamic estimates were calculated as

$$\%\;\text{error}=100\times \frac{\left(\Delta C_{\mathrm{HbO}/R}^{\text{measured}}-\Delta C_{\mathrm{HbO}/R}^{\text{simulated}}\right)}{\Delta C_{\mathrm{HbO}/R}^{\text{simulated}}},$$

where $\Delta C_{\mathrm{HbO}}^{\text{simulated}}=50\;\mu M$ and $\Delta C_{\mathrm{HbR}}^{\text{simulated}}=-0.05\;\mu M$.

**Fig. 8:**
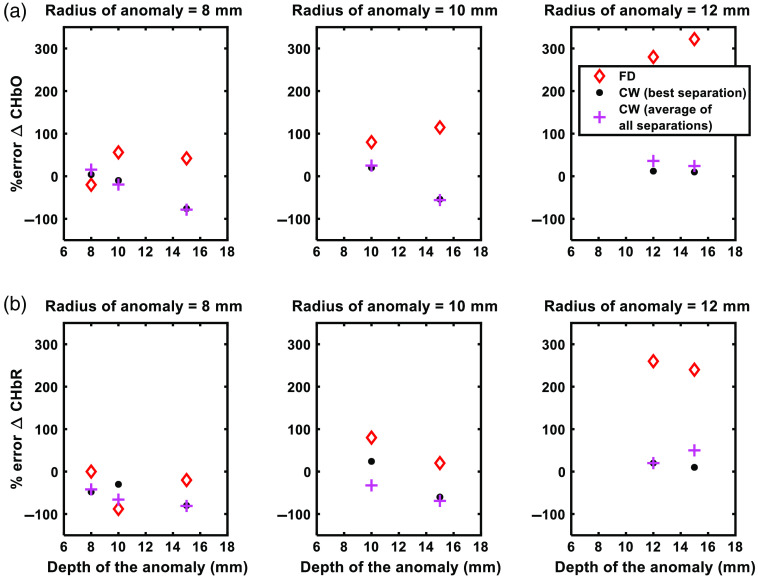
Results of seven finite-element simulations of function activation showing % error in estimations of (a) oxygenated and (b) deoxygenated hemoglobin. Red diamonds represent results from multi-distance FD NIRS, blue dots represent results from CW-NIRS (best source–detector separation, i.e., source–detector separation that yielded highest response), and magenta crosses represent results from CW-NIRS (average of all source–detector separations).

Figure 8 summarizes the average error in estimation of hemodynamic changes [Fig. 8(a): % error in $\Delta C_{\mathrm{HbO}}$ estimates and Fig. 8(b): % error in $\Delta C_{\mathrm{HbR}}$ estimates], for anomalies of different sizes (each column) and at different depths (x axis of each plot). In each plot, the red diamonds represent results from multi-distance FD NIRS, blue dots represent results from CW-NIRS (best source–detector separation, i.e., source–detector separation that yielded highest response), and magenta crosses represent results from CW-NIRS (average of all source–detector separations). We can observe that the error in estimated changes of the concentrations increase with anomaly size and depth in FD results. Anomalies that were at shallow depths likely had uniform sampling of the perturbation by all source–detector separations, lessening the impact of the partial volume effect. By contrast, the concentration changes estimated with MBL law were relatively constant. Note that, although the error in estimated concentration changes is lower with CW-NIRS, the overall magnitude is still high. This could be because the presence of the anomalies could violate the inherent assumption of homogenous media in both MBL and FD-NIRS analyses. With FD-NIRS, our results show that the use of multiple source–detector separations in the analysis contributes to errors to a larger extent.

## 4 Discussion

The availability of commercial fNIRS devices, the standardization of data processing methods,^47^ and the development of algorithms to correct for motion artifacts^48^^–^^52^ or extra-cerebral hemodynamic changes^53^^,^^54^ have helped expand functional activation measurements to challenging experiments such as auditory stimulation—in which the activation regions are expected to be 2 to 3 cm below the surface of the head. However, most functional activation experiments—with some exceptions^26^^,^^55^—still use CW approaches despite the availability of commercial instruments that can implement the frequency domain approach. Indeed, this is the first study to use a frequency domain approach to record hemodynamic changes due to functional activation of the auditory cortex. Our primary objective was to compare the more popular CW approach with the more rigorous FD approach.

Using the CW-NIRS approach, we measured a 0.1≈0.2  μM change in concentration of oxygenated hemoglobin, which is consistent with similar CW measurements in the literature.^7^^,^^18^^,^^32^ For example, Chen et al.,^7^ Issa et al.,^18^ and Wiggins et al.^32^ observed a maximum $C_{\mathrm{HbO}}$ change of 0.4, 0.1, and 0.2  μM, respectively. With the FD-NIRS approach, however, we measured a consistently higher change in concentration of oxygenated hemoglobin. Both CW and frequency domain results were obtained from the same data set, albeit using different models of photon diffusion to account for tissue scattering effects. The CW approach analyzes intensity measurements at each source–detector separation and approximates scattering effects with the MBL law. The FD approach directly measures the tissue scattering coefficient ($\mu_{s}^{\prime }$) with photon fluence measurements at multiple source–detector separations. The multi-distance approach inherently assumes that measurements made at different source–detector separations are equally sensitive to changes in tissue optical properties—this assumption is often invalidated in functional activation measurements that feature focal hemodynamic changes. In this study, multi-distance FD measurements of the photon fluence rate were performed at source–detector separations of 1.6 to 4.5 cm; longer separations are sensitive to deeper tissue depths. Thus a focal change in tissue absorption at say 10 to 15 mm below the surface will elicit a greater change in photon fluence rates at 2.5 cm source–detector separation than 1.5 cm. This partial volume effect could explain why the FD-NIRS analysis systematically overestimates hemodynamic response to functional activation. To verify our results, we performed controlled finite-element simulations of the functional activation process. Our simulation results confirm that, due to partial volume effects, heterogeneous/focal tissue oxygenation changes, such as those due to functional activation, are better measured with single-distance rather than multi-distance approaches.

We note a few limitations of our comparison. Despite our results suggesting that FD-NIRS approaches can overestimate functional activation hemodynamics, frequency domain instruments remain superior to simpler CW-NIRS instruments because they can directly measure scattering effects. One limitation of this study is that it compared CW-NIRS with multi-distance FD approaches. Some or all of the biases introduced by partial volume effects could potentially be addressed by newer frequency domain instruments that operate at a single source–detector separation^56^^,^^57^ or fast look-up table inversion methods for optical property estimation.^3^ These approaches typically solve for the photon diffusion equation at multiple modulation frequencies, which are less susceptible to partial volume effects. Comparison of single-distance CW-NIRS with single-distance FD-NIRS is an important future study. A second limitation is the use of homogeneous tissue models for the analysis of functional activation hemodynamics. Extra cerebral flow/oxygenation changes can significantly confound functional activation measurements.^53^ Here we have partially mitigated these effects by only using measurements at a longer source–detector separation for FD-NIRS analysis. A better approach would be to use multi-layered tissue diffusion models.^58^^–^^60^ Another potential solution to address the partial volume effects is to use time-domain approaches^61^ that use optical measurements from one source–detector pair to fully solve the photon diffusion equation. However, because they need specialized light sources and complex instrumentation, TD instruments are not commercially available and are less common in the clinic. A more practical alternative is diffuse optical tomography, which uses multiple single-distance CW diffuse optical measurements to create a 2D/3D map of tissue oxygenation changes. Notably, Culver et al.^62^ showed that accurate determination of functional activation changes can be achieved by reconstructing an image of tissue hemodynamic changes from a plurality of single-distance CW diffuse optical measurements.

## 5 Conclusion

Functional stimulation of the auditory cortex was studied using CW-NIRS and FD-NIRS approaches and their results were compared. The responses from multi-distance FD-NIRS yielded baseline measurements, whereas the CW-NIRS approach estimated the changes in the concentration of HbO and HbR. For functional activation changes, i.e., focal changes, CW-NIRS offered better estimation of changes in concentration of HbO and HbR, compared with that of the multi-distance FD-NIRS analysis. The results obtained suggest that the single-distance MBL analysis approach is well suited for measuring changes due to functional activation stimulations.
