# Supplementary material for: Comparison of functional activation responses from the auditory cortex derived using multi-distance frequency domain and continuous wave near-infrared spectroscopy
Source: Neurophotonics. 2021 Dec 15;8(4):045004. doi: 10.1117/1.NPh.8.4.045004 (PMC8673635; doi:10.1117/1.NPh.8.4.045004)
Supplement: Supplementary file 1 [file NPh_008_045004_SD001.pdf]

SUPPLEMENTRY FIGURES

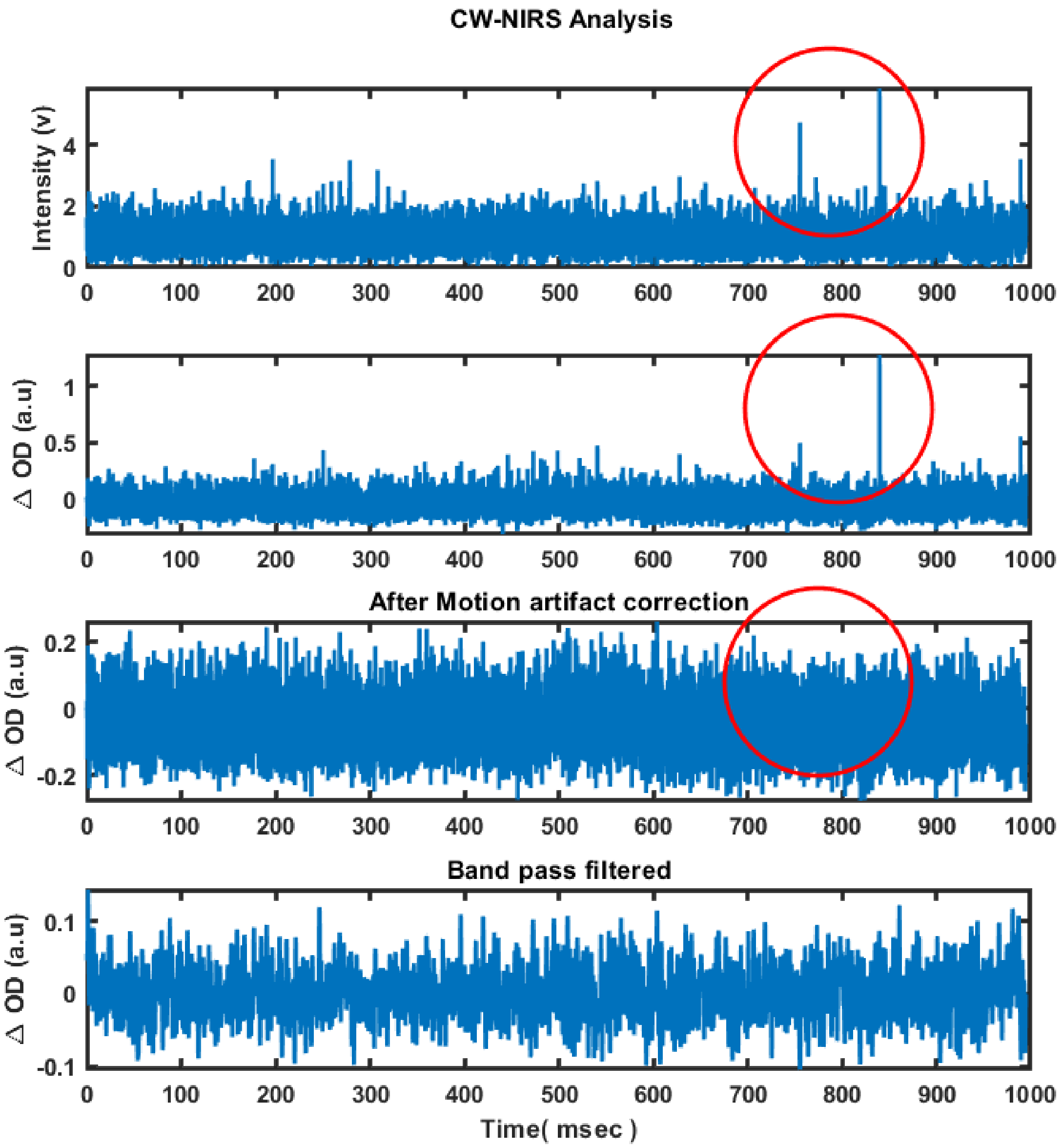

Figure 1 CW-NIRS Analysis performed over each subject data. The first and second figures show the raw intensity and the optical density(Δ OD) at a single source detector position.Motion artifact correction is performed over the ΔOD.The MA corrected data is then bandpass filtered(0.01 Hz - 0.5 Hz) and is shown in the fourth figure.

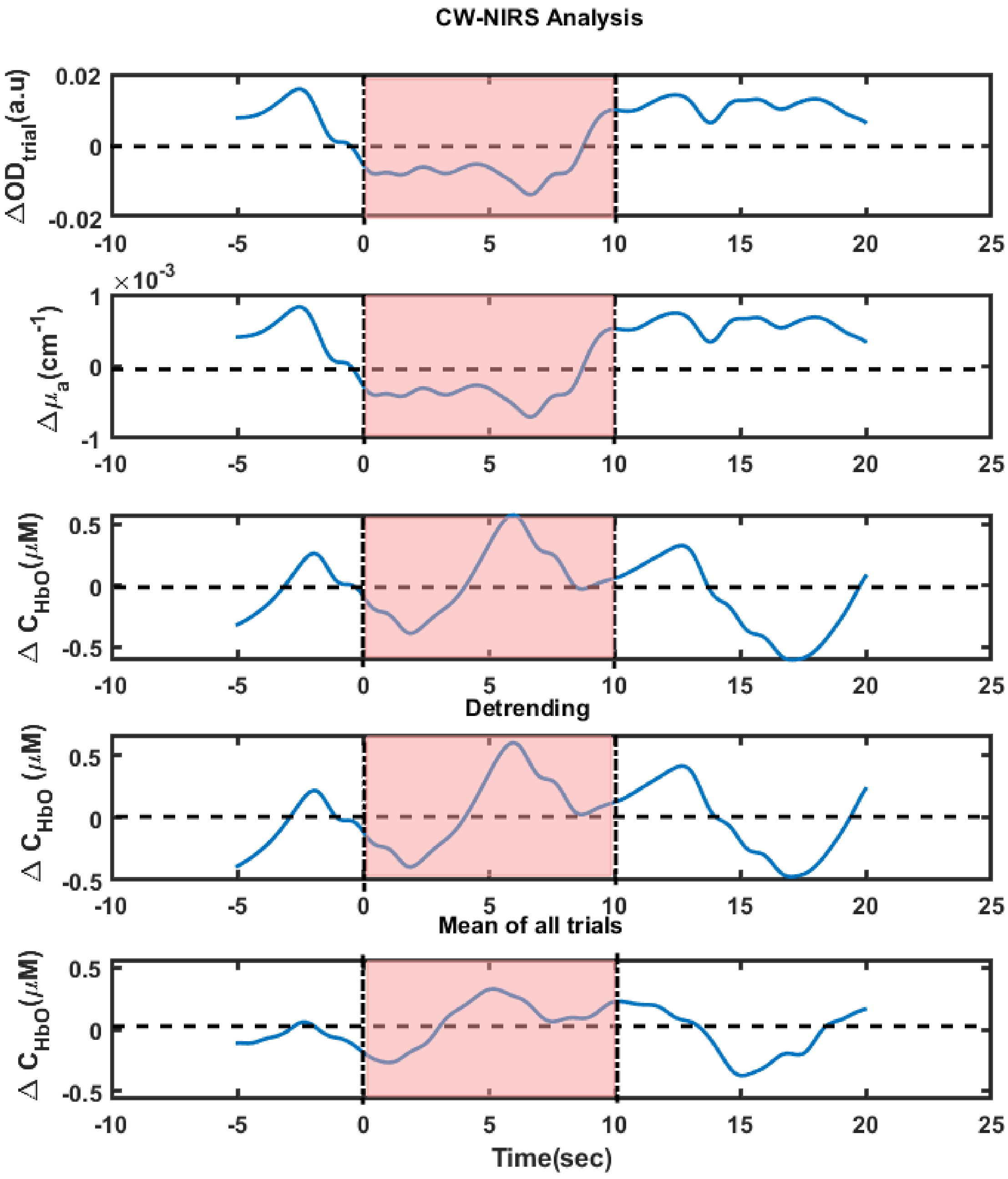

Figure 2 The figures show a trial of 25 second epoch from a subject at a single source detector separation. The first figure shows a Δ OD from one epoch. The second and third figures show the conversion of ΔOD to $\Delta\mu_a$ and to ΔC_{HbO}. Detrending is performed over the obtained ΔC_{HbO}. A mean response across all trials is shown in the last figure.

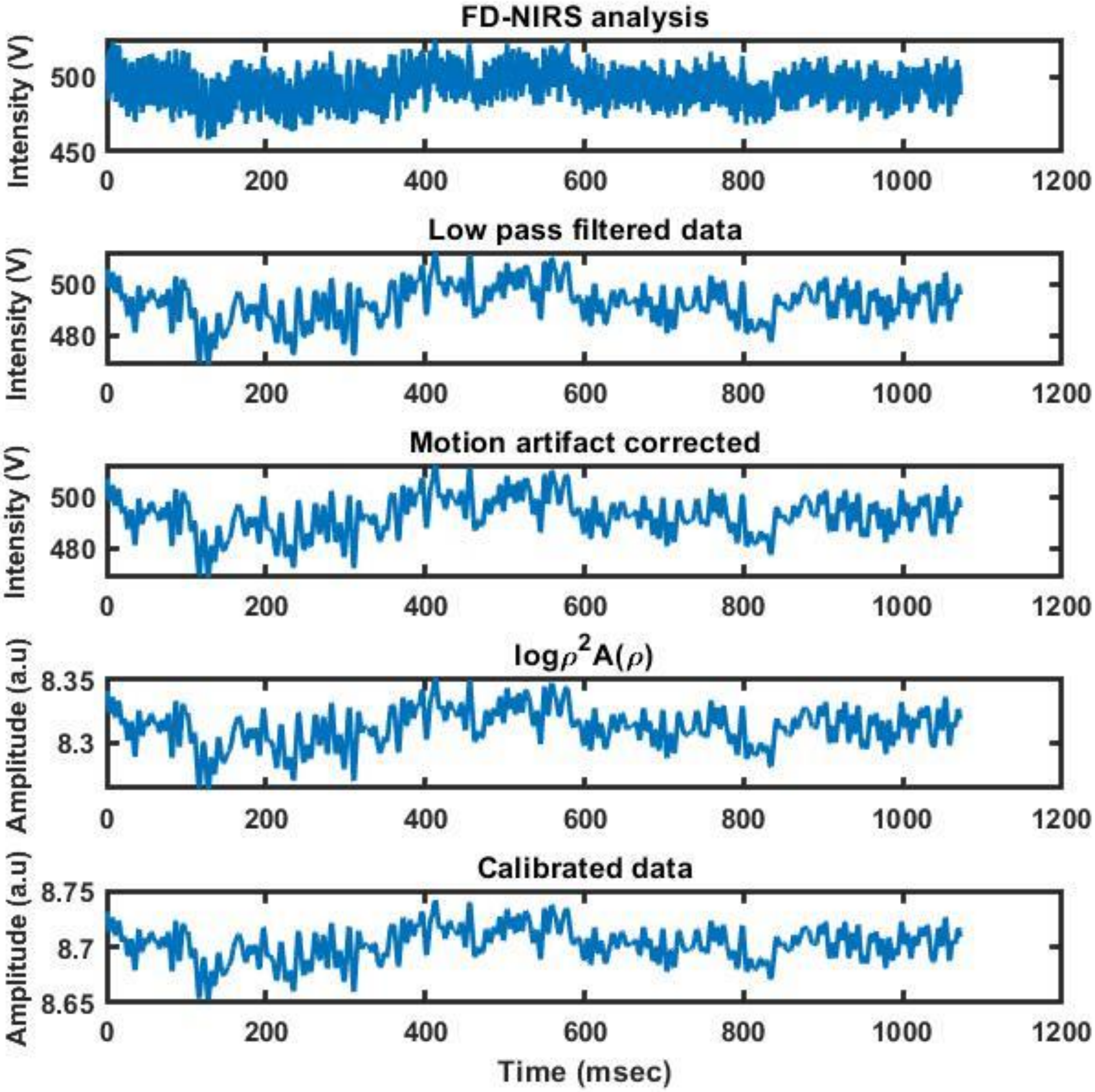

Figure 3 The figure shows FD-NIRS analysis performed over a single subject. The first and second figures show the raw intensity at a single detector position. The second and third figures show the low pass filtered and motion artifact corrected data. The third and fourth figures show the Amplitude $(log\left(\rho^2\left(A(\rho)\right)\right))$ *. A calibration is performed on the amplitude data as shown in the fifth figure.*

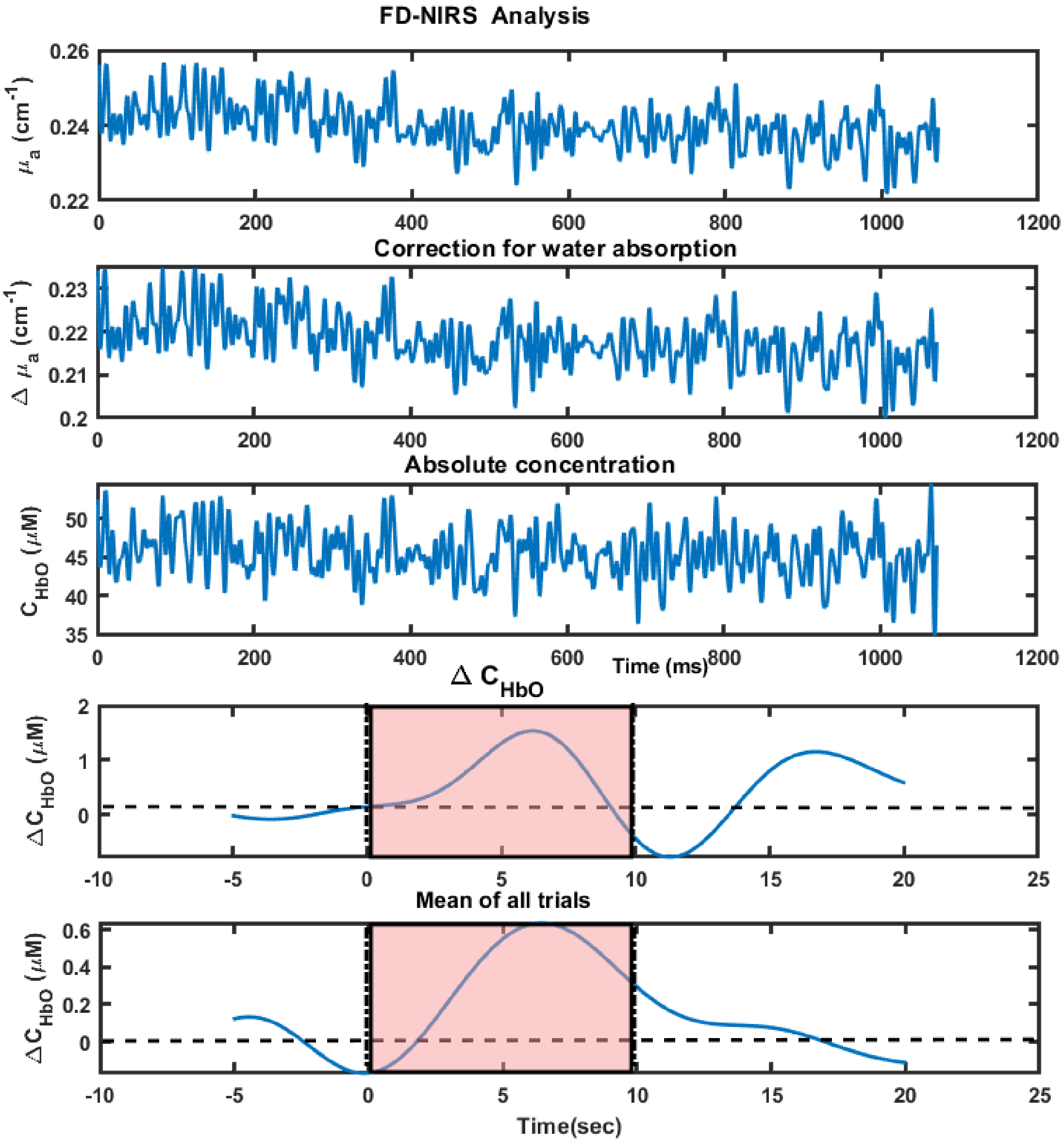

Figure 4 The figure shows the data analysis steps performed after the data is calibrated. The calibrated data is then fit to obtain the κ_i and κ_r values which are the converted to get obtain the absorption coefficients. The μ_a and μ_{sp} are then corrected for water absorption as seen in second figure. The absolute concentration is measured from μ_a and μ_{sp} in figure three, which is then divided into epochs and the baseline concentration is subtracted to measure $\Delta CHbO$ in figure four. All the epochs are averaged to obtain the response from a single detector seen in figure five.
